# Atomic Layering, Intermixing and Switching Mechanism in Ge-Sb-Te based Chalcogenide Superlattices

**DOI:** 10.1038/srep37325

**Published:** 2016-11-17

**Authors:** Xiaoming Yu, John Robertson

**Affiliations:** 1 Engineering Dept, University of Cambridge, Cambridge CB2 1PZ, UK

## Abstract

GeSbTe-based chalcogenide superlattice (CSLs) phase-change memories consist of GeSbTe layer blocks separated by van der Waals bonding gaps. Recent high resolution electron microscopy found two types of disorder in CSLs, a chemical disorder within individual layers, and SbTe bilayer stacking faults connecting one block to an adjacent block which allows individual block heights to vary. The disorder requires a generalization of the previous switching models developed for CSL systems. Density functional calculations are used to describe the stability of various types of intra-layer disorder, how the block heights can vary by means of SbTe-based stacking faults and using a vacancy-mediated kink motion, and also to understand the nature of the switching process in more chemically disordered CSLs.

Ge,Sb,Te-based phase change materials are the basis of electronic non-volatile storage-class memories that are a major contender to replace Flash memories^1^,^2^,^3^,^4^. The present versions use a rapid structural transition between their bulk crystalline and amorphous phases. However, it is desired to reduce the switching energy of these devices. One possibility is to use superlattices, whose interfaces cause more phonon scattering, thereby reducing their thermal conductivity and lowering the energy needed to reach a particular switching temperature^5^. A further development of this idea is to form ‘interfacial phase change memories’ or ‘chalcogenide superlattices (CSL)’ with atomic scale periodicities so that the transition might occur between two different crystalline phases of the CSL at a lower energy cost^6^,^7^,^8^,^9^,^10^,^11^,^12^,^13^.

Such short period Ge,Sb,Te CSLs have been fabricated and electrical switching between two resistance states has been demonstrated with lower switching energy^6^,^10^. There have been various suggestions as to how the atomic switching process occurs in these CSLs, such as by electric field, carrier injection, or thermal activation, but it is not yet agreed. This is partly because the exact atomic structures of the low- and high-resistance states of the CSLs is not agreed. To understand this, Fig. 1 shows the four low energy configurations for the shortest period CSLs based on nine chemically ordered layers in each x,y plane. The layers are labeled A, B, C according to a hexagonal stacking sequence along the z axis. They have the strong bonds within each layer aligned to retain resonant bonding between the layers. Other possible arrangements based on chemically ordered layers have higher total energies, see Fig. S1 in the supplementary information, taken from Table 1 of ref. 14.

These limiting models and high resolution scanning transmission microscope (STEM) images^8^,^13^ led to two slightly different switching models for the CSLs. In one case, Tominaga *et al.*^7^,^8^,^9^ suggested a transition between the Ferro (F) and the Inverted-Petrov (IP) states, with a flipping of one set of GeTe bilayers. On the other hand, Takaura *et al.*^10^,^11^,^12^,^13^ suggested a transition between the Petrov (P) and Inverted-Petrov states, with a flipping of two GeTe bilayers. Yu and Robertson^14^ modeled the overall transition and noted that both cases would be a two-step process with a Ge-Te flip along the hexagonal z axis plus a lateral movement within the layers in the x,y plane to regain the lower energy states. They calculated a switching energy barrier for both models of about 2.5–2.7 eV, close to the experiment value^12^. Thus, the CSL switching process was modeled as a change of stacking sequence in chemically pure layers, without any change in atomic coordination numbers^14^,^15^,^16^. It differs from the local displacive transition of the crystalline to amorphous phase transition in bulk GeSbTe systems^17^,^18^, where the Ge coordination number changes, and from the thermally driven process of the thicker superlattice devices^5^. Note that there are no 4-fold Ge sites in the cells of Fig. 1.

Subsequently, the Z-contrast STEM images of Momand *et al.*^19^ on switchable CSL samples suggest that CSLs are less ordered structures than the simple models of Fig. 1, and thus the switching process must be more complex. The STEM images showed that the superlattices form Ge,Sb,Te blocks separated by van der Waals (vdW) bonding gaps, and that the blocks could be thicker than the 9 atomic layers. The images suggest that the number of layers in individual blocks could vary by the motion of atomic bilayers similar to a stacking fault, connecting one block to another^19^,^20^. These bilayers seem to consist of Sb-Te bilayers from the Z contrast. The bilayer connections were seen by other groups^14^,^21^, suggesting a general low energy defect of the system.

The Z contrast STEM images^19^ and extended X-ray fine structure (EXAFS) spectra^22^ found that the atomic layers were more chemically mixed than the basic models of Fig. 1, as in some structural models of bulk Ge_2_Sb_2_Te_5_^23^,^24^,^25^. The layers particularly near the vdW gap tend to have a mixed (Ge,Sb)Te composition. Now, the previously proposed switching mechanisms^7^,^13^,^14^ involved switching within pure GeTe bilayers. This may be a problem, as Sb enrichment of these layers would tend to suppress the Peierls distortion of GeTe bilayers^26^,^27^ that creates the bistable states to switch between. Thus the observed structures may be less switchable. To resolve these issues, we consider the effect of disorder on CSL stability, the processes enabling disorder, and finally possible switching processes in more disordered CSLs.

## Calculations and Discussion

**Figure 1:**
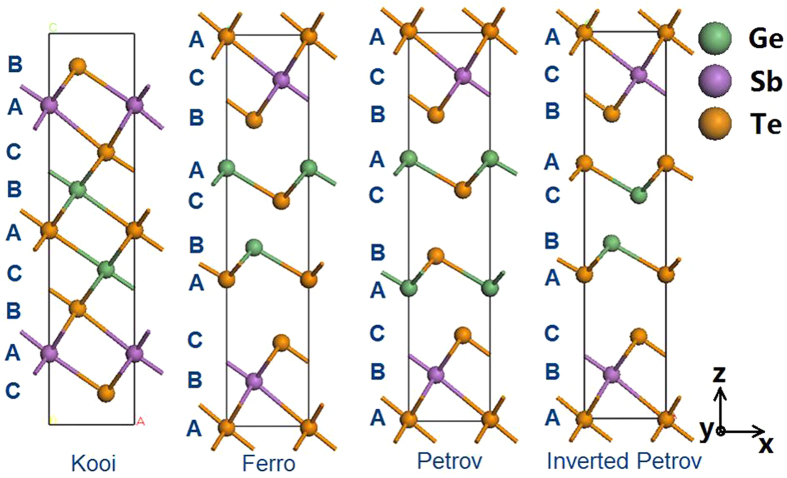
Four basic Ge_2_Sb_2_Te_5_ superlattice structures, labeled with hexagonal stacking. Green spheres = Ge, purple spheres = Sb, Orange spheres = Te.

We have calculated the total energy per cell for the structures of Fig. 1 at 0 K by density functional theory using the CASTEP code^28^ and using various density functionals for the electronic exchange-correlation energy, as given in Table 1. The simplest functional is the generalized gradient approximation (GGA)^29^. GGA does not treat van der Waals bonding correctly, so we also used two correction schemes to GGA, that of Grimme^30^ and that of Tkatchenko and Scheffler^31^,^32^. The main purpose of the vdW correction schemes is to give vdW bond lengths closer to experimental values; they do not shift relative energies much compared to GGA itself. We also include in Table 1 the total energies calculated by the Heyd-Scuseria-Erzenhof (HSE) hybrid functional^33^, as an example of including a fraction of exact exchange in the exchange-correlation functional. Finally, we list GGA total energies that include spin-orbit coupling (SOC)^7^. Table 1 shows that the lowest energy structure for chemically ordered layers is that of Kooi^24^ in both GGA and the vdW-corrected GGA schemes. On the other hand, the hybrid functional gives a different ordering of energies, as also found by Sosso *et al.*^26^.

**Figure 2:**
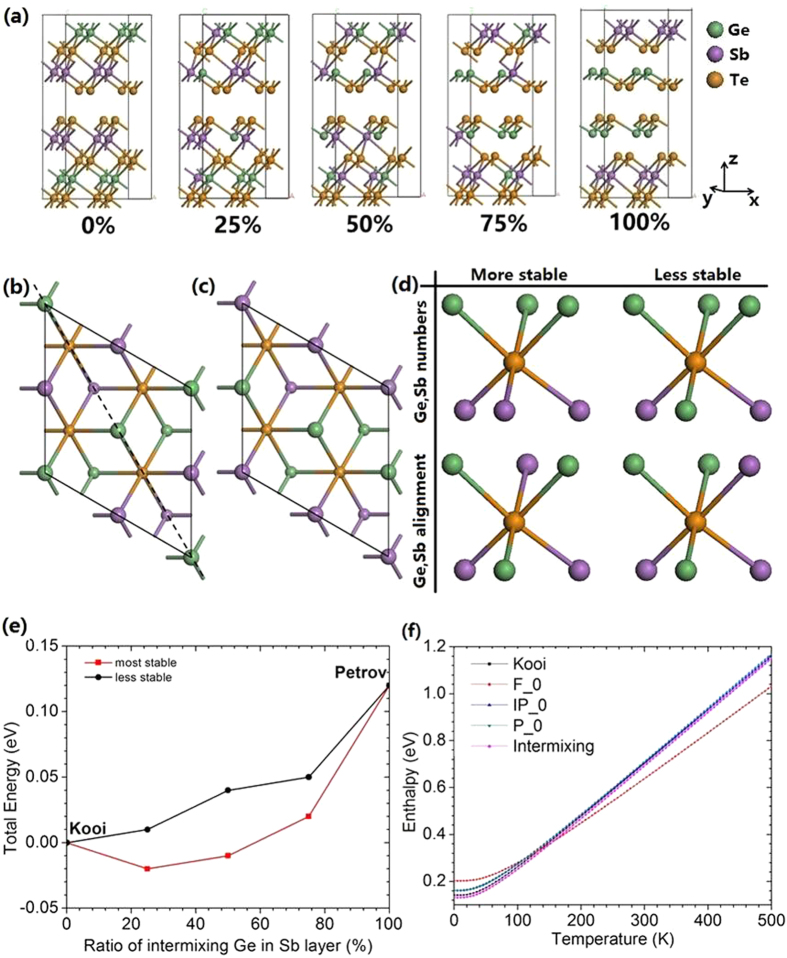
(**a**) Five GST hexagonal blocks with different Ge:Sb mixing ratios ranging from 0% to 100%. Green spheres = Ge, purple spheres = Sb, Orange spheres = Te. (**b,c**) Top view of two different orderings of mixed Ge,Sb layers; (**b**) with a mirror plane (shown as dashed line), and (**c**) without. Large balls are atoms on the top layer. (**d**) Ordering factors that affect the total energy. (**e**) Relative total energy per formula unit for the various atomic arrangements. Note that one mixed structure has a lower energy than Kooi. However, the energy of the mixed structure depends strongly on how the mixing occurs, between the cases (**b,c**). (**f**) Variation of enthalpy with temperature.

We now consider the effect of chemical disorder within the layers. We make a 2 × 2 × 1 supercell of the 9-layer Kooi structure and disorder the atoms in the Ge and Sb layers to 0%, 25%, 50%, 75% and 100% towards the Petrov structure, Fig. 2(a). The structures were relaxed internally, to allow them to acquire Peierls distortions. The total energies are plotted against mixing ratio in Fig. 2(e), and in Fig. S3.

We find that their total energy depends strongly on how the intra-layer disordering is carried out. Figure 2(b,c) shows top views of two limiting cases of intra-layer ordering in the 2 × 2 cell. Considering three layers of Sb,Te,Ge. If the 50% mixing case has an internal mirror symmetry as in Fig. 2(b), then the structure is more stable, and the total energy vs ordering bows down to a lower energy at the mixed composition as in the red line in Fig. 2(e). If the 50% mixed structure shows no mirror symmetry as in Fig. 2(c), the structure is less stable, and the total energy varies more linearly between the Kooi and Petrov values as in the black line in Fig. 2(e). The 50% mixed structure with mirror symmetry of Fig. 2(b) is essentially that of Matsunaga *et al.*^23^, and it has a lower energy than the Kooi structure in GGA-TS, as also found by Sosso *et al.*^26^. It possesses a Peierls distortion so that its bond lengths (Fig. S2) are close to those observed experimentally^23^,^26^, unlike those of the Kooi structure.

The dependence of total energy on the type of intra-layer ordering follows the rules previously given by Da Silva *et al.*^27^. These rules are summarized in Fig. 2(d). The more stable structures are those in which a central Te site is surrounded by three Ge neighbors and three Sb neighbors, their so-called 3Ge-Te-3Sb rule. The second rule is that the more stable structures have Ge sites and Sb sites on opposite sides of the octahedral Te sites, rather than a Ge-Te-Ge or Sb-Te-Sb alignment across the Te site.

It turns out these rules are the same as those derived from the vacancy model of Wuttig *et al.*^33^. These authors noted that a structure is stabilized if the local composition and fraction of structural vacancies (equivalent to van der Waals gaps) leads to a half-filled p-band in each orthogonal direction. This band occupation also maximizes the amount of Peierls distortion. DaSilva^27^ expressed the condition for a maximum Peierls distortion in terms of the difference in atomic radii of Ge and Sb, but it is also the half-filled band condition due to the different valences.

The effect of finite temperatures and lattice vibrations on the enthalpies of these structures is calculated by density functional perturbation theory (DFPT). The lattice vibrations cause the enthalpies of the Kooi and other structures to rise more quickly with temperature than for the Ferro structure, Fig. 2(f). The dependence for the mixed structures is similar to that of the Kooi and Petrov structures, so that the cross-over to Ferro is shifted to lower temperature. This effect can be used to preferentially grow non-Kooi configurations at lower temperatures^8^.

**Figure 3:**
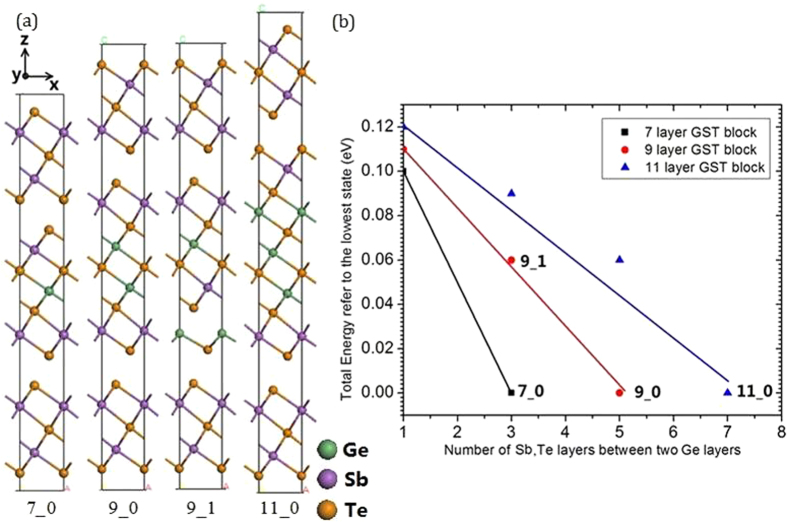
(**a**) 7-, 9- and 11- layer GST blocks with Sb_2_Te_3_ separation layers, showing two examples of 9-layer case. The first number is the total number of layers in the middle, the second number is the number of SbTe bilayers between the two GeTe units. (**b**) Relative total energies vs. number of Sb,Te layers between the two Ge layers within the GST block. Total energy decreases as the spacing of the Ge layers decreases, due to the GeTe layer moving away from the van der Waals gap. Green spheres = Ge, purple spheres = Sb, Orange spheres = Te.

The Z contrast STEM images of Momand *et al.*^19^ show that many GST superlattices have an odd number of layers per block, and tend to have Sb-rich layers next to the vdW gaps. In order to understand these larger blocks, we have calculated the total energies of GST blocks with different atomic stackings. Figure 3 shows the relative energies at 0 K of 7, 9, and 11 layer GST blocks containing additional GeTe layers, as a function of the number of GeTe layers between the two SbTe bilayers. Typical structures are illustrated in Fig. 3(a). Each GST block is separated by a Sb_2_Te_3_ block to minimize inter-block interactions via the outer layers. The most stable stackings for a certain block size have been marked as 7_0, 9_0 and 11_0. Their total energy is adjusted to 0 eV for comparison. (The first number is the total number of layers; the second number is the number of SbTe bilayers between the two GeTe units). For better understanding, the 9_1 is shown in Fig. 3. To demonstrate its difference to 9_0, we see that 9_0 has no SbTe layers between the two GeTe layers, whereas 9_1 has one SbTe bilayer between the two GeTe layers. We see that the blocks with the lowest energy at 0 K are those with the SbTe bilayers at the edge of each block, facing the vdW gap. This is consistent with the more stable bulk GST structures^20^,^24^.

**Figure 4:**
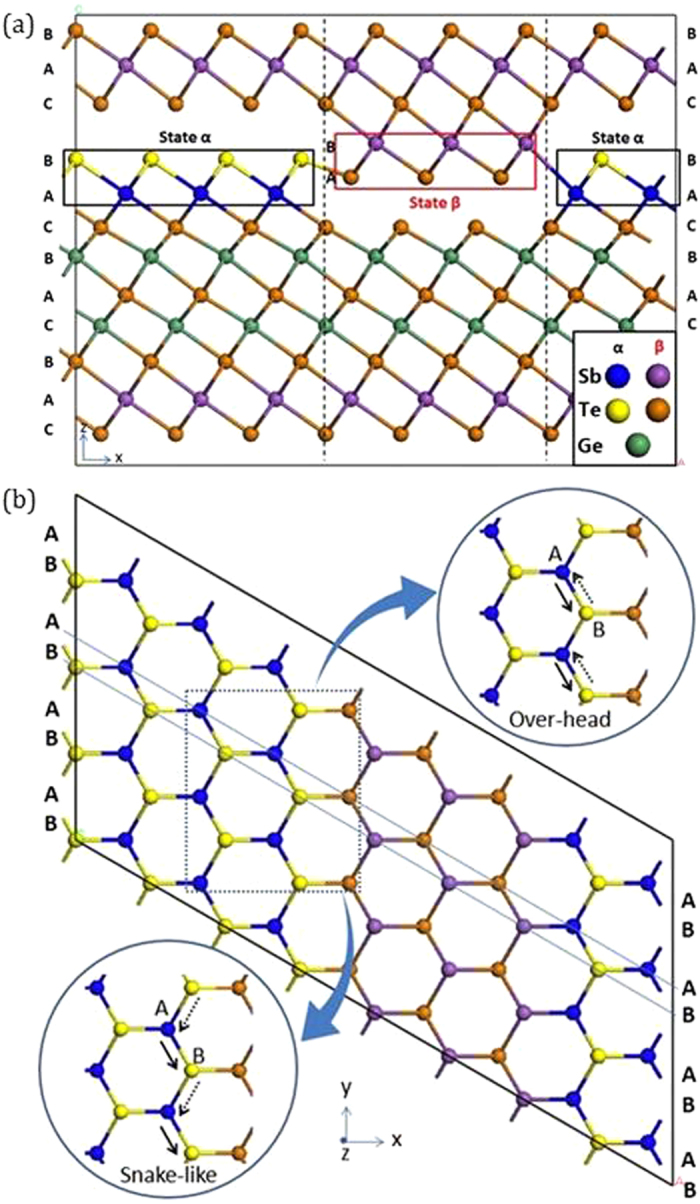
(**a**) Side view of vdW gap boundary model. The Sb-Te bi-layers are highlighted in the rectangles. In state α, Sb is blue and Te is yellow. In state β, Sb is purple and Te is orange. States α and β are connected by like-atom bonds. (**b**) Top view of over-head and snake-like switching process. The Sb in the site A swaps with Te at site B. Due to the two movement directions of Te, it creates two possible path ways. The dotted square highlights the area where switching happens.

We now consider the mechanism of changing of block heights by the stacking fault defects. The bilayer stacking faults are seen when the samples are annealed to 300 °C^19^,^20^, which sets a typical activation energy for the process. To study the stacking faults, we take a 9-layer block of the Kooi structure and create an 8 × 1 × 1 supercell as in Fig. 4(a). We then add a further SbTe bilayer on its top to form an 11 layer block, so that the vdW gap is now between the SbTe bilayer and the next Kooi-like block. Layers in this phase at the edge of the model are denoted state α and are also labeled according to the hexagonal stacking sequence.

Three bilayer atom pairs in the centre of the primitive cell are then flipped so that the Sb-Te bilayer detaches from the lower block and attaches to the upper block to form the stacking fault; this is denoted state β in Fig. 4(a). The flipped atoms in state β must align their stacking to those in the upper block. This requires that the Sb and Te layers exchange their vertical stacking sequence, to give a Te-Sb-Te-Sb-Te stacking. Second, the Sb and Te sites must also shift laterally to align their bonds with those in the upper block. It is not just a vertical motion. Overall, the transition is Sb_A_ + Te_B_ → Sb_B_ + Te_A_ from state α to state β. Due to a lack of symmetry, the left hand boundary of state β differs from that on the right. The left hand boundary consists of Te-Te like-atom bonds, whereas the right hand boundary consists of (weak) Sb-Sb bonds.

These transitions could occur in three ways. The first is an ‘overhead rotation’ of a complete column of Sb-Te sites in Fig. 4(b), similar to that in the previous study^14^. We calculated an energy barrier for this process to be ~3.3 eV, which is large compared to values of ~1.75 eV expected for a process that occurs at around 300 °C (using the equation E = kT × ln(νt) to relate energies to characteristic temperatures for thermal excitation, where ν is an attempt frequency of ~10^13^ Hz and t is an observational time of ~100 s^34^). A second possibility is a ‘snake-like’ motion of the atoms along the boundary line which collectively exchanges the positions of the Sb and the Te sites, as in Fig. 4(b). We calculate that this process has a much lower energy barrier of 1.6 eV. This lower energy arises because each atom movement breaks only one bond per site not two^14^.

**Figure 5:**
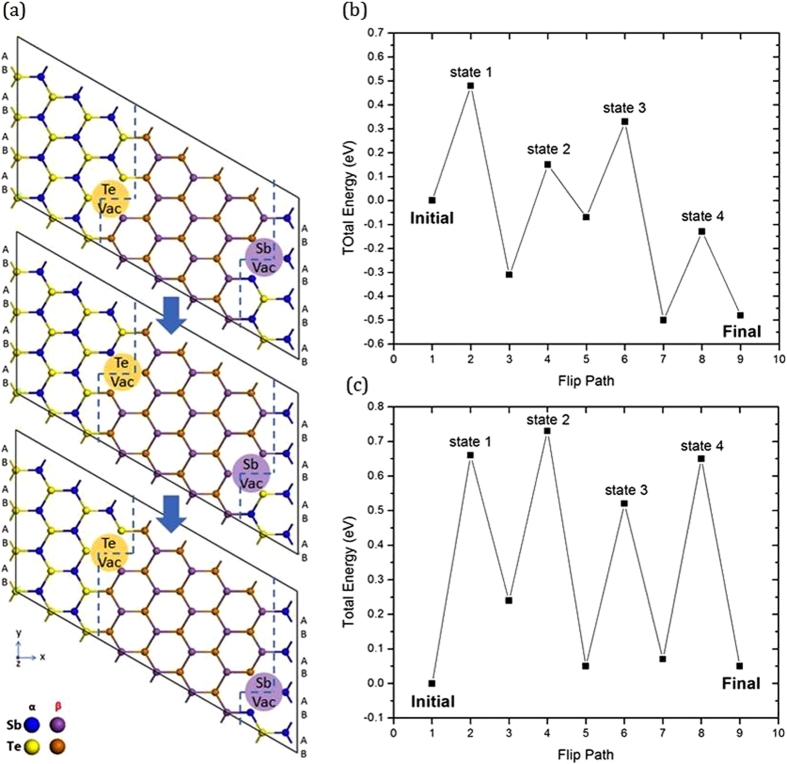
(**a**) Top view of vdW gap boundary model, showing the up movement of Te vacancy along the left-hand boundary, allowing atoms to move from state α to state β. It is similar for the right-hand boundary. (**b,c**) Energy diagram of transition states during vacancy movement in SbTe bilayer, for left-hand and right-hand boundary respectively.

The third possibility is to introduce a vacancy at the boundary line which then moves along the boundary line as a ‘kink’, exchanging atoms one by one from state α to state β, Sb_A_ → Sb_B_ and Te_B_ → Te_A_. Electronic vacancies have interesting roles in GeSbTe compounds^35^,^36^. The movements on each boundary are shown in Fig. 5(a). On the left side, a Te vacancy moves along the boundary, and allows each atom (Sb or Te) to jump from state α to a more stable site in the state β. The Te vacancy alternates as a Sb_Te_ antisite at each step. After the vacancy has passed along the line, the atoms from state α have moved to state β. On the right side, we move a Sb vacancy rather than a Te vacancy as the cost of a Sb vacancy is lower than a Te vacancy there. The transition energy states for both boundaries are shown in Fig. 5(b,c). (The effect of different functionals is shown in Fig. S4. The effect of using the nudged elastic band method to calculate barrier heights is shown in Fig. S5). State 1, 2, 3 and 4 are marked as the transition states with the highest energy during the movement of the vacancy. Both flip processes (left and right boundaries) show a small energy barrier (0.5–0.7 eV) compared to collective motion, so the possibility of stacking fault movement can be very high at low temperatures of 250–300 °C. A Te vacancy costs ~1.9 eV to form. A Sb vacancy costs 1.43 to 1.69 eV. Thus the total cost is about 2.3 eV via the vacancy mechanism.

**Figure 6:**
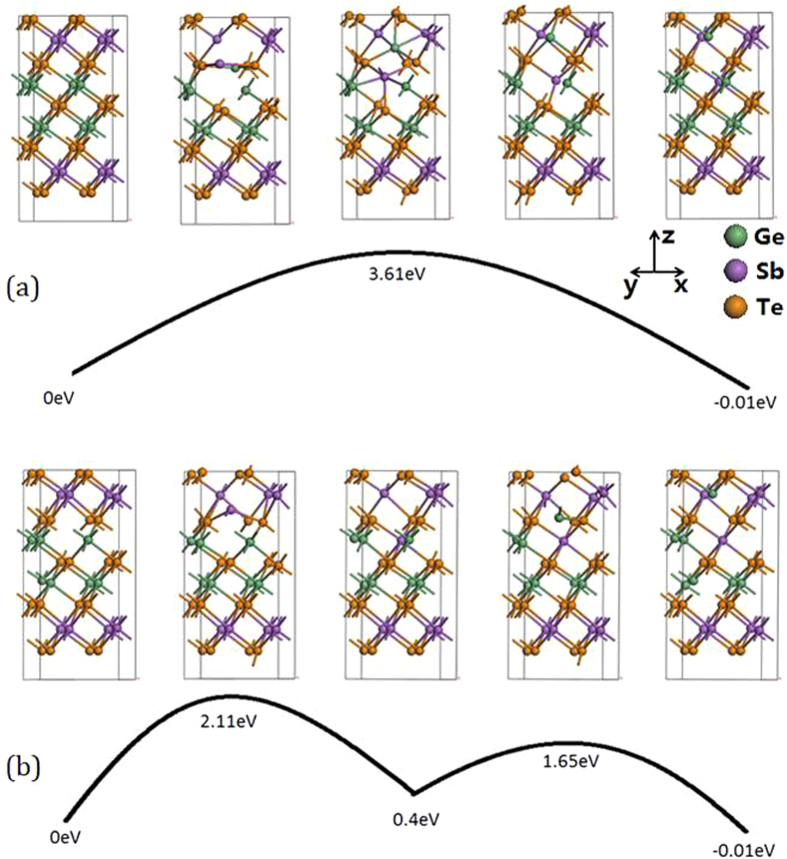
(**a**) Atomic exchange without a vacancy. From left to right: one Sb and one Ge exchange by a rotational pathway. The energy barrier is 3.61 eV. (**b**) Atomic exchange with a Ge vacancy. From left to right: one Sb moves down to a Ge vacancy site and leaves a Sb vacancy at the upper layer; then one Ge moves upwards and sits in the Sb vacancy. The energy barrier is 2.11 eV. Green spheres = Ge, purple spheres = Sb, Orange spheres = Te.

We now consider the kinetic stability of individual atoms within CSLs, in terms of vacancy migration in the Ge,Sb sublattices. We first calculate the barrier for the direct exchange of Ge and Sb atoms for the path in Fig. 6(a). There are sufficient voids in the CSL structure for this exchange to occur by a rotational path. But the calculated barrier of 3.61 eV is large. The second path uses an existing Ge vacancy, as in Fig. 6(b). After being occupied by a Sb atom, it leaves a new vacancy in the Sb layer. Then a Ge atom migrates to this site and creates a new vacancy. This whole process keeps the vacancy at the original layer, but exchanges the two Sb and Ge atoms with each other. The barrier is now lowered to 2.11 eV. The formation energy of the Ge vacancy is ~0.9 eV for the Ferro or Inverted Petrov structure but only 0.38 eV for the Petrov phase. Thus, the total diffusion barrier is of order 2.5–3.0 eV which is still quite high. This energy barrier is similar to that found by Deringer^37^. Thus, the CSLs are moderately stable against diffusive mixing.

We finally consider the atomic switching mechanism, if the layers are more chemically mixed as in the models of Fig. 2(a). The GeTe lattice has shorter and longer bonds, differing in length by 16% due to the Peierls distortion, it supports two stable configurations to switch between. In contrast, the Sb_2_Te_3_ lattice has two bond lengths differing by only 1.3%. It has a center of symmetry, with the shorter bonds in the middle and the longer bonds on the outside. This centre of symmetry means that this structure does not support bistable states. We note that the Kooi structure does not switch because it also has a centre of symmetry.

**Figure 7:**
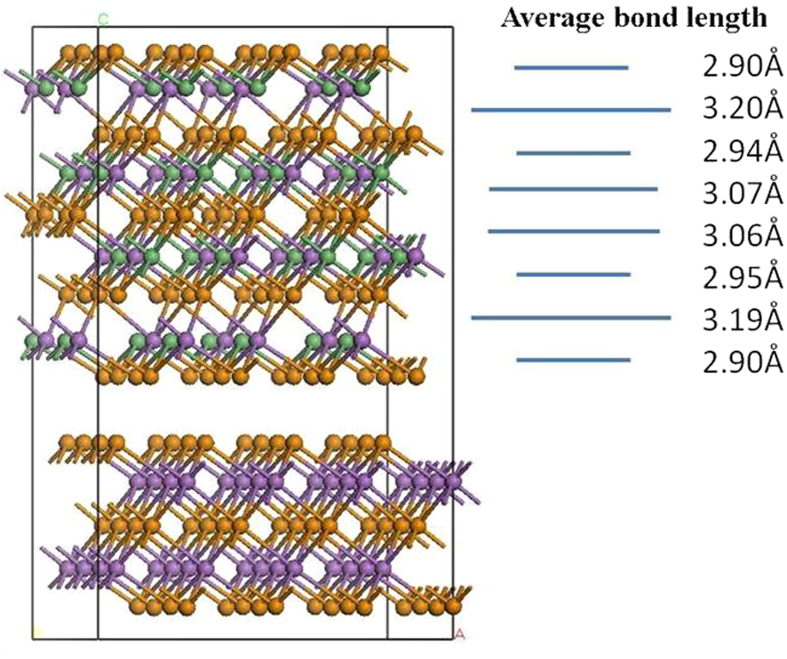
Bond lengths in the 50:50% mixed structure of Fig. 2(b), separated by an inert Sb_2_Te_3_ block. This shows the large Peierls distortions, which are similar to experimental values. Green spheres = Ge, purple spheres = Sb, Orange spheres = Te.

**Figure 8:**
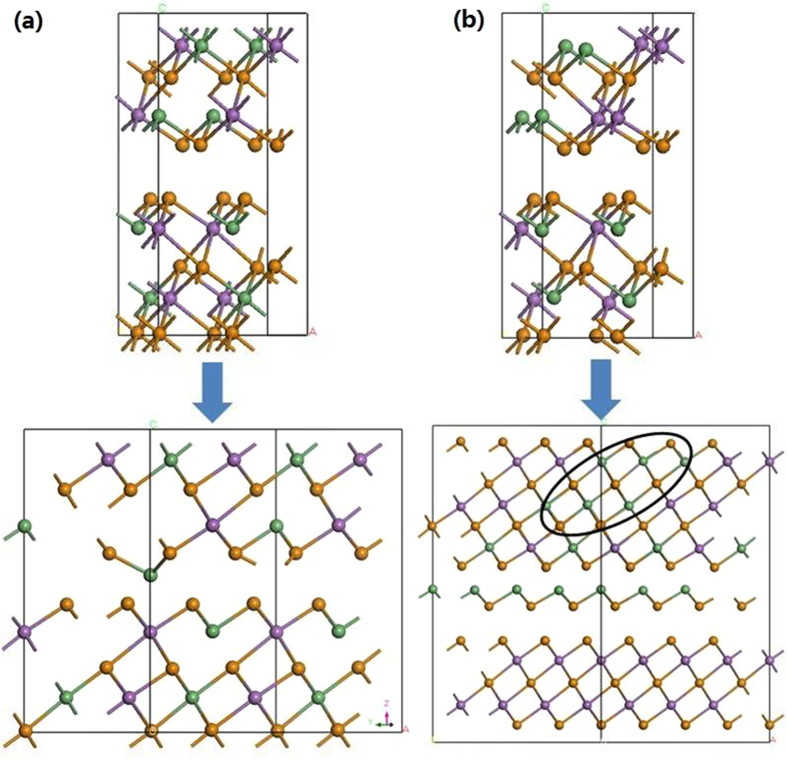
(**a,b**) Switching in two different models of mixed layer GST blocks. (**a**) follows the structure of Fig. 2(b) and has Peierls distortions, enabling it to switch as in a chemically ordered structure, or locally by local flips as in ref. 15. (**b**) does not possess a strong Peierls distortion and will not switch, unless it has local Ge-rich clusters of at least 4 Ge sites (circled), which then allow local Peierls distortions enabling a form of switching. Green balls = Ge, purple balls = Sb, Orange balls = Te.

Analysis of the various possible structures gives two cases. Figure 7 shows the first case of a supercell of the more stable of the chemically disordered structure from Fig. 2(b). It shows a strong Peierls distortion with notably short and long bonds. These arise from the half filled p-bands. This structure has two metastable states with the short and long bonds reversed. In the limiting case, the switching between these states can occur on an atom by atom basis, as in Fig. 8(a), following the recent simulation of Kalikka *et al.*^15^.

In the second case, Fig. 8(b), for the less stable version of intra-layer disordering as in Fig. 2(c), the Peierls distortion is weak and does not occur in all cases. We now find that there must be some Ge-clustering so that locally there can be sufficient Peierls distortions to provide the local metastable states. In this case, clustering of three or more Ge-Te units within the layers is required to allow a Peierls distortion and to define two different local structures to switch between.

Overall, these conditions will allow a switching mechanism based primarily on aligned vertical flips of GeTe layers to occur, as proposed by optical pump-probe experiments^9^. Thus, the source of the reduced switching energy for CSL can still remain a phase transition between two solid polytypes over a shorter distance and in reduced dimensions. However, the driving force for the transitions is not settled, between an electric field effect, charge injection, or a more thermally driven effect. Scaling relations between the current and the SET resistance as seen in bulk devices^4^ may argue in favour of a thermal mechanism^38^.

## Conclusions

A previous paper^14^ described a possible switching mechanism for chalcogenide superlattice memories, assuming their atomic structure consisted of idealized chemically order layers of fixed height, with GeTe bilayers adjacent to the van de Waals bonding gap, as originally proposed^7^,^13^. In that case, switching involved the flipping of GeTe bilayers, with no change in the Ge coordination number. Subsequent Z contrast STEM images and EXAFs data suggested that the CSLs were more chemically disordered and the GST blocks had variable height. We have used density functional calculations to understand the energetics of disordering, and to retain as much as possible of the previous switching mechanism in the presence of disordering. The GST blocks are found to vary their heights by SbTe bilayers passing as stacking faults between adjacent GST blocks, which cost only 1 eV plus a vacancy formation energy. The activation energy for atomic diffusion between layers using vacancies is found to be quite large, ~2.5 to 3.0 eV including vacancy formation energy, favoring thermal stability of the CSLs. It is found that the more stable forms of disordering still have Peierls distortions, and so allow switching between their metastable states.

## Methods

### Ab initio simulations

The calculations used the plane wave, density function theory (DFT) CASTEP code^28^ using ultrasoft pseudopotentials. The plane waves represent the valence electrons Ge 4 s^2^ 4p^2^, Sb 5 s^2^ 5p^3^ and Te 5 s^2^ 5p^4^. The exchange correlation functional uses the generalized gradient approximation (GGA) of Perdew-Burke-Ernzerhof (PBE)^29^. Spin-orbit coupling is not included. The van der Waals correction is added to the GGA using the Tkatchenko/Scheffler^31^,^32^ scheme with the DFT-D2 correction function. This takes into consideration the vdW contributions of atoms due to their local chemical environment. The Grimme^30^ scheme is also used for reference. The plane-wave cut-off energy is set to 400 eV for all the calculations. For the primitive cells in Figs 1 and 3, a 7 × 7 × 1 Monkhorst-Pack grid is used. A sparser Kpoint grid (3 × 3 × 1) is chosen for the 2 × 2 supercells in Fig. 2. The same K-point is used to get the energy barrier in Fig. 6. For calculating the transition states of models in Figs 4 and 5, 1 × 3 × 1 Kpoint grid is used. For the cluster models in Fig. 7, a Gamma point with an origin shift (0.25 0.25 0.25) is used. All models have been fully relaxed and the total energy is converged to less than 1 × 10^−6^ eV per atom. The value for acceptable residual force is 0.03 eV Å^−1^ and the stress tolerance is 0.05 GPa. The thermodynamic properties such as enthalpy are calculated from the phonon dispersion simulation using density functional perturbation theory (DFPT)^39^,^40^. To be detailed, linear response with interpolation is used. The q-vector grid spacing for interpolation is 0.05/Angstrom. Convergence tolerance is 10^−5^ eV/Ǻ^2^. The separation for dispersion is 0.015/Ǻ. For energy barrier calculations, we use the transition state search algorithm. The complete linear synchronous transitions (LST) and quadratic synchronous transitions (QST) simulation was used to find the transition states^41^. The energy barrier of collective motion in Fig. 4(b) is found directly from the atomic pathway between initial and final states, while the multi transition states of Fig. 5(b/c) are calculated by dividing the pathway of the vacancy into steps, Fig. 5(a). The transition states were recalculated using the TS confirmation tool from CASTEP which is based on the Nudged Elastic Band method (NEB)^41^,^42^, and which is confirmed by the synchronous transit method^43^.

## Additional Information

**How to cite this article**: Yu, X. and Robertson, J. Atomic Layering, Intermixing and Switching Mechanism in Ge-Sb-Te based Chalcogenide Superlattices. *Sci. Rep.* **6**, 37325; doi: 10.1038/srep37325 (2016).

**Publisher’s note**: Springer Nature remains neutral with regard to jurisdictional claims in published maps and institutional affiliations.

## Supplementary Material

Supplementary Information

## Figures and Tables

**Table 1:** Total energy of the four ordered structures calculated by different exchange-correlation functions^30^,^31^,^44^.

	GGA (no vdW)	GGA + TS	GGA + Grimme	with SOC^7^	HSE
Kooi	0.00	0.00	0.00	−0.17	0.11
Ferro	0.06	0.03	0.05	0.22	0.09
Petrov	0.22	0.12	0.20	0.06	0.13
Inverted Petrov	0.15	0.21	0.09	0.00	0.00
