# Supplementary material for: Atomic Layering, Intermixing and Switching Mechanism in Ge-Sb-Te based Chalcogenide Superlattices
Source: Sci Rep. 2016 Nov 17;6:37325. doi: 10.1038/srep37325 (PMC5112535; doi:10.1038/srep37325)
Supplement: Supplementary Information [file srep37325-s1.doc]

**Supplementary Information for ‘Atomic Layering, Intermixing and Switching Mechanism in Ge-Sb-Te based Chalcogenide Superlattices’**

Xiaoming Yu and John Robertson

Engineering Dept, University of Cambridge, Cambridge CB2 1PZ, UK


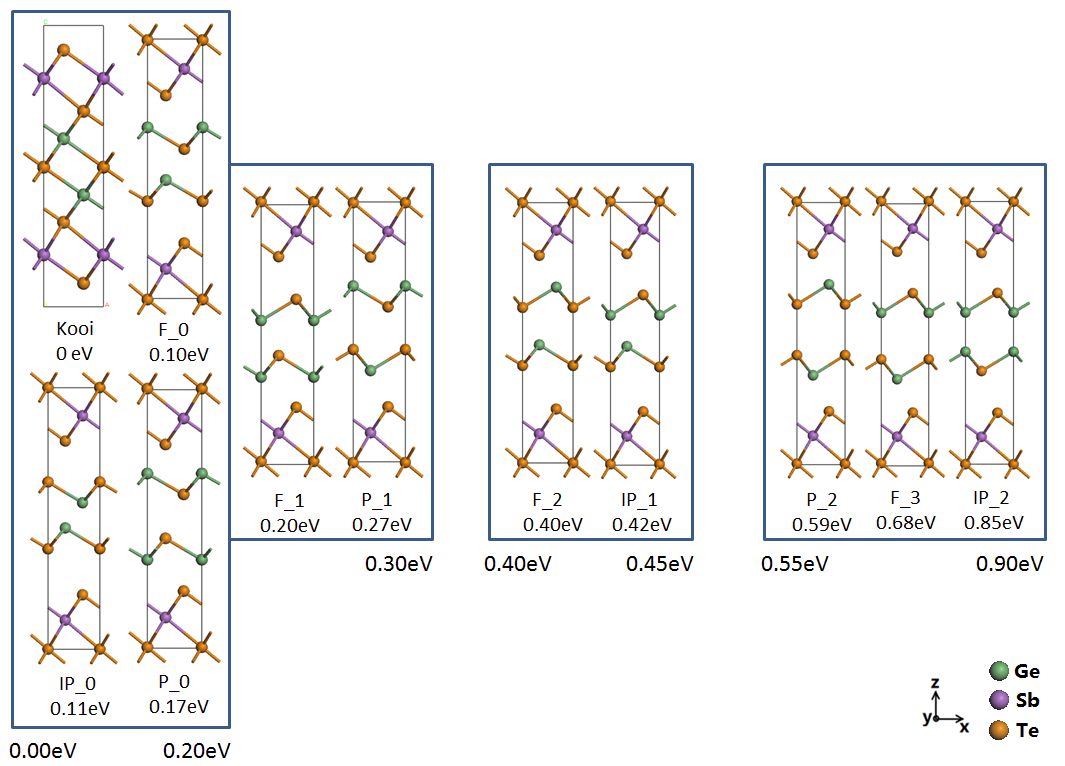


Fig. S1. Illustration of how the four basic structures of Fig. 1 in the main text, shown here on the left, have a much lower calculated energy at 0K. Other variants, labelled as in ref [14] and grouped into blocks of similar energy range, have strong bonds not aligned between layers to favor resonant bonding and have higher energies. Ferro = F, Petrov = P Inverted Petrov = IP.


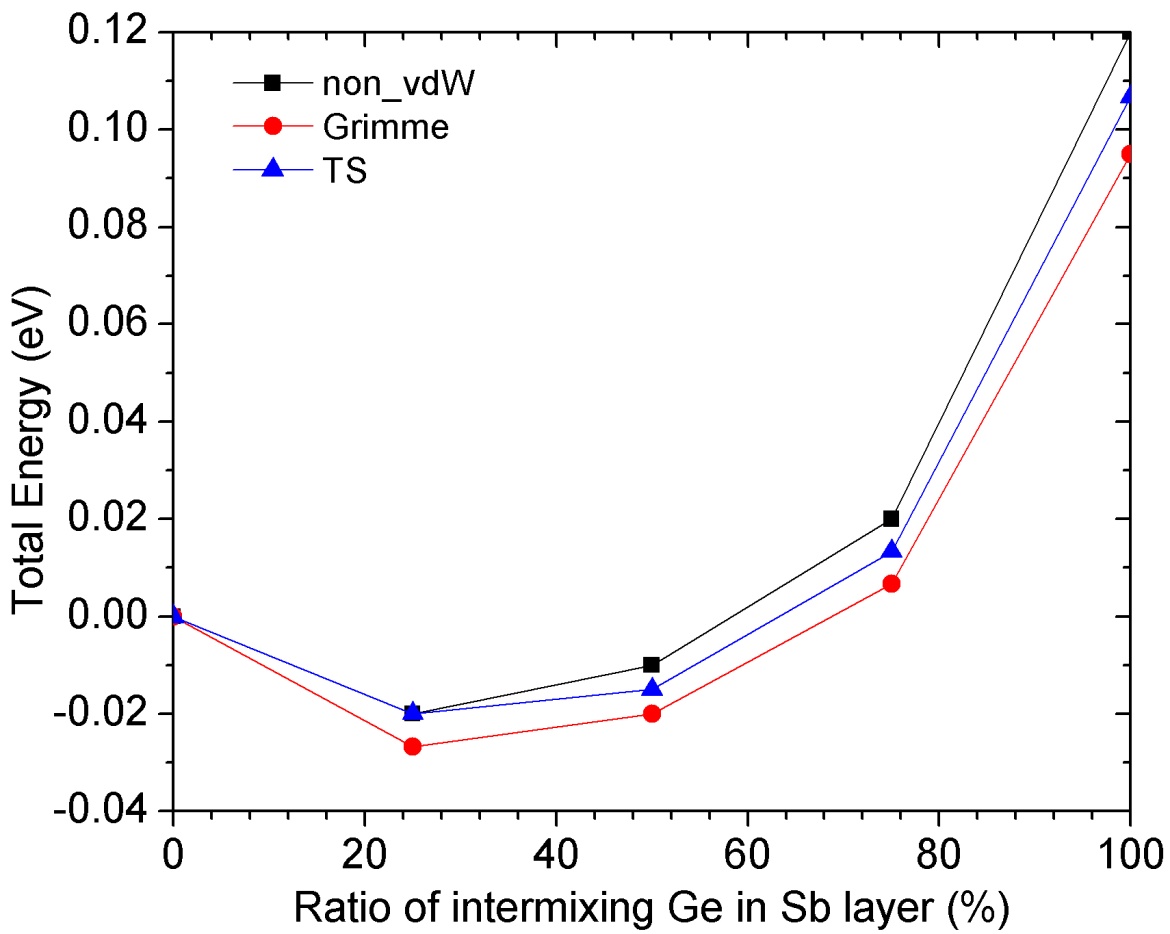


Fig. S2. Comparison of total energy vs vdW functional for the intermixed model (2*2 supercell)


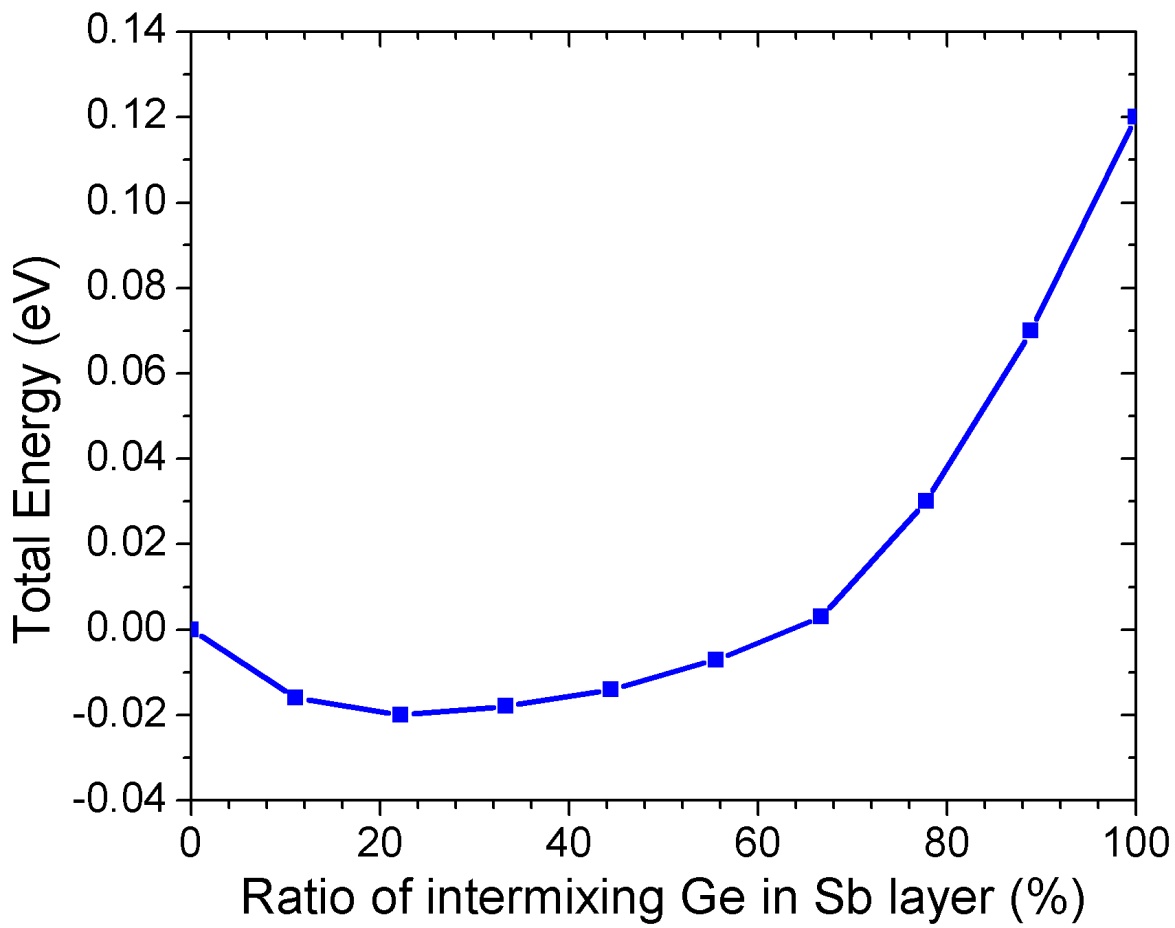


Fig. S3. Total energy per formula unit vs mixing ratio for a 3x3 supercell. To be compared to Fig 2(e) for a 2x2 cell. As there are 9 atoms in a layer, the ratio is changed gradually by moving one Sb/Ge each time.


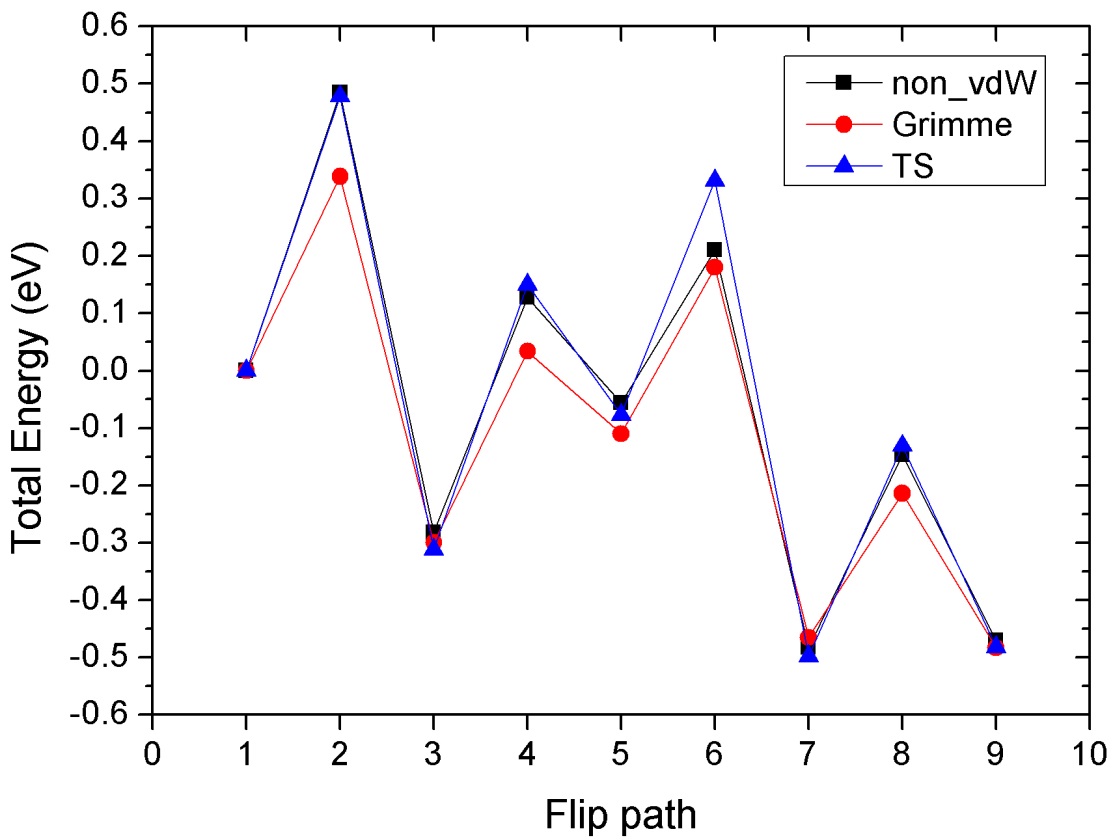


Fig. S4. This is the test for the vdW correction on the vacancy movement of Fig 5(b). The results are given by the complete linear synchronous transitions (LST) and quadratic synchronous transitions (QST) in CASTEP.


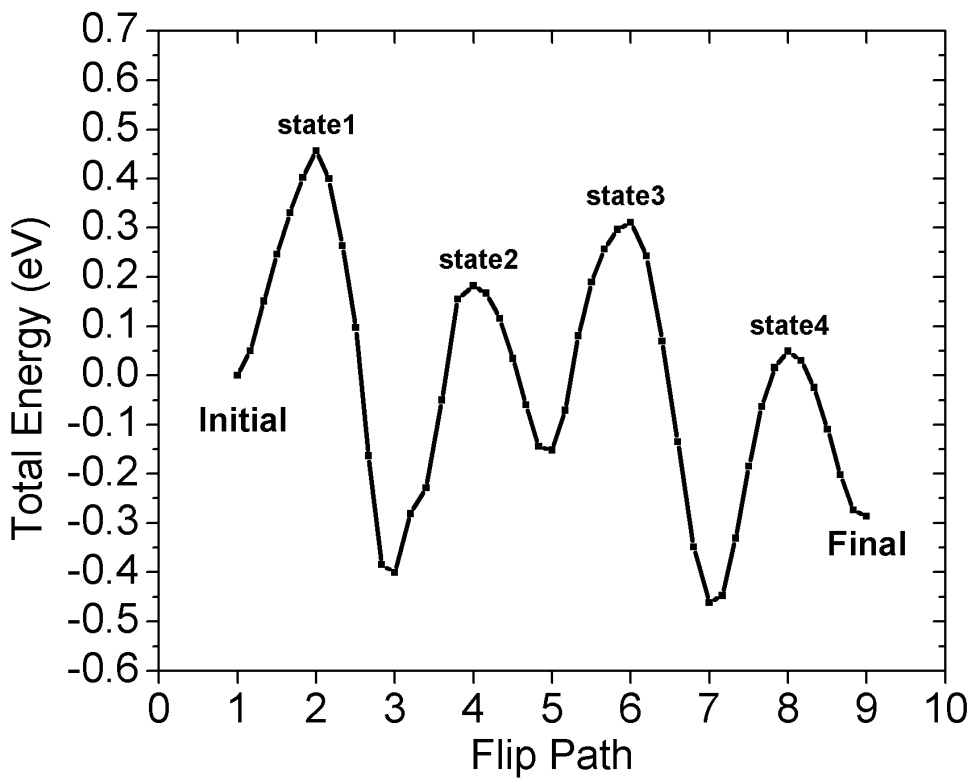


Fig. S5. This plot compares the barrier energies given by the NEB method to those from the LST method in Fig S4, using the same functional and pseudopotential.
